# Sexual risk behavior and substance use among young, diverse women seeking care at a reproductive health clinic

**DOI:** 10.1186/s12905-019-0709-2

**Published:** 2019-01-21

**Authors:** Alyssa L. Norris, Carla Rich, Naomi Krieger, Kate M. Guthrie, Clair Kaplan, Kate B. Carey, Michael P. Carey

**Affiliations:** 1 Centers for Behavioral and Preventive Medicine, The Miriam Hospital, Coro West, Suite 309, 164 Summit Ave, Providence, RI 02906 USA; 2 Department of Psychiatry and Human Behavior, Alpert School of Medicine, Brown University, Providence, RI 02906 USA; 3 Department of Clinical Research, Planned Parenthood of Southern New England, 345 Whitney Avenue, New Haven, CT 06511 USA; 4 Center for Interdisciplinary Research on AIDS, Yale University, 135 College Street, Suite 200, New Haven, CT 06510 USA; 5 Department of Behavioral and Social Sciences, Brown University School of Public Health, 121 S. Main Street, Providence, RI 02903 USA; 6 Center for Alcohol and Addiction Studies, Brown University, 121 S. Main Street, Box G-S121-5, Providence, RI 02912 USA

**Keywords:** Sexual health, Reproductive health centers, Young adult women

## Abstract

**Background:**

To describe sexual risk behavior, alcohol (and other substance) use, and perceived health promotion needs among young adult women seeking care from an urban reproductive health care clinic in the Northeastern United States, and to examine if these needs differ by race and ethnicity.

**Methods:**

Women 18–29 years old presenting for a routine medical visit were invited to participate. Of 486 eligible women, 466 (96%) agreed to participate and completed a brief survey on a tablet computer. Most of the sample (53%) identified as non-Hispanic White. One-quarter (25%) identified as Hispanic/Latina. A smaller proportion of women identified as African American (19%).

**Results:**

One-third (31%) of women reported a history of sexually transmitted infection (STI), and women reported infrequent condom use with recent sexual partners. Regarding behavioral health needs, nearly three-quarters of women (72%) reported regular alcohol use, approximately one-third had used marijuana (37%) or tobacco (33%) in the last month, and 19% reported clinically significant depressive symptoms in the last two weeks. Women reported moderate-to-strong interest in receiving information about relationships and sexual health; however, the majority were not interested in information about their substance use. Hispanic and African-American women were more likely to report STI history despite reporting fewer sexual partners than non-Hispanic White women. Minority women also reported significantly less alcohol and cigarette use, but more water pipe tobacco use, and reported significantly greater interest in interventions to promote sexual health. Hispanic women also evidenced significantly elevated rates of depressive symptoms, with 26% of Hispanic women reporting a clinically significant level of depressive symptoms.

**Conclusions:**

Reproductive health centers are opportune settings to address a broad range of healthcare needs, including sexual health, substance use, and mental health. These centers engage a diverse group of women, which is important given observed disparities in health outcomes based on race/ethnicity. Young women, particularly racial and ethnic minority women, report the most interest in services addressing sexual and relationship health.

Young women, particularly racial and ethnic minority women, are particularly vulnerable to sexually transmitted infections (STIs), accounting for a disproportionate percentage of all newly acquired STIs in the United States [1]. Young women evince the highest incidence of chlamydia, gonorrhea, and human papillomavirus (HPV) infection [1]; and one-quarter of all 20 to 24-year-old women acquire HPV annually [2]. Despite representing only 13% of the U. S. population, African-American women account for 62% of new HIV infections among women and have 2-to-10 times the rates of reported chlamydia, syphilis, and gonorrhea cases compared to White women [3]. Although African-American young adults have the highest rates of these STIs of any racial/ethnic and age categories, Hispanic individuals also have elevated risk compared to non-Hispanic Whites [3]. Young women are also uniquely at risk for unintended pregnancy [4] and sexual assault [5]. Consequently, sexual and reproductive health care is especially important for this age group and for particular subgroups of women.

Perhaps because of their unique sexual health vulnerability, young women often consider their gynecologist to be their primary care provider and their most trusted medical professional [6]. Among women in the U. S. aged 15–24, the majority (59%) sought reproductive health services in the past year [7]. Given women’s reliance on and trust in sexual health providers, reproductive health centers are well-positioned to offer not only family planning, STI prevention and treatment, general gynecologic care and sexual assault triage, but also to address young women’s broader health needs. This is particularly important given that integrating STI prevention services with outpatient medical and behavioral health care is an effective way to reduce sexual risk intentions and behavior compared to traditional, “siloed” care [8, 9]. This may be especially important for minority women, as Hispanic and African-American adults have low rates of mental health service utilization despite equivalent or higher rates of mental health concerns [10].

Therefore, it is important to identify the needs of young adult women presenting for care in an effort to provide comprehensive services, and also to identify any racial/ethnic differences in these needs. Two important correlates of risky sexual behavior are alcohol use and depressive symptoms. For many young women, alcohol use often co-occurs with sexual behavior; research has documented alcohol use as an important correlate of sexual initiation, unprotected sex, and risky sexual behavior [11]. More than half of women aged 18 to 44 drink alcohol, and young women aged 18 to 24 have the highest rates of “binge drinking” (≥ 4 drinks during one drinking episode) relative to other age cohorts [12]. One in five sexually active high school females reported using alcohol during their last sexual encounter [13], and heavy drinking in particular is associated with having sexual intercourse [14]. Although research suggests that White women are more likely than Hispanic and African-American women to be current drinkers [15], other research suggests that there are no racial/ethnic differences in heavy drinking among women who drink [16]. Therefore, it is important to understand how substance use and sexual risk align among a diverse group of women. Similarly, rates of depressive symptoms are increasing among young women [17]. This is important for women seeking medical care at reproductive clinics, because depressive symptoms are associated with young women’s risky sexual behavior, such as a greater number of sexual partners and substance use prior to last sexual encounter [18, 19]. Further, it is important to note that although Hispanic, Non-Hispanic White, and Non-Hispanic Black women experience similar rates of depression [20], African-American and Hispanic adults are less likely to receive any treatment for depression [21].

In summary, young women are (a) at elevated risk for STIs and unintended pregnancies, (b) often use alcohol before sex, (c) evince concerning increases in depressive symptoms, (d) rely on reproductive health centers for their healthcare needs, and (e) benefit from integrated health services. Despite these facts, screening for substance use and misuse in reproductive health care settings remains uncommon. This is unfortunate because screening for substance use as well as sexual risk behavior in reproductive health care settings affords an opportunity to improve young women’s health, in line with recommendations for screening, brief interventions, and referral for substance use treatment within ambulatory health care settings [22]. Leading gynecological providers therefore recommend such screening become an integral part of women’s health visits [23].

With the goal of improving this aspect of young women’s care, we sought to better understand young women’s needs and preferences regarding behavioral health services. Therefore, the purpose of this study was threefold; in the context of a reproductive health setting, we describe young women’s sexual behavior, mental health, and substance use, associations between alcohol and risky sex, and interest in behavioral intervention to guide future health promotion services. Moreover, given racial and ethnic disparities in health (e.g., STIs, alcohol use) and health treatment, we examine these outcomes based on race and ethnicity. Although the overarching purpose of this study was exploratory to establish the needs of young women presenting for care, we hypothesized that Hispanic and African-American women would have higher rates of STIs and be less likely to engage in alcohol misuse compared to White women, given population-level disparities.

## Material and methods

The Institutional Review Board approved all procedures. This research took place at an urban, community-based, reproductive health care clinic located in the northeastern U. S. where routine women’s health care is provided by nurse practitioners, nurse midwives and physician assistants.

### Procedures

Potential participants were initially identified through medical chart reviews of patients presenting to the clinic. English-speaking patients in the eligible age range (18 to 29 years; *N* = 531) were called from the waiting room to a private room where a research assistant obtained verbal consent, confirmed their age, and invited them to answer a screening survey on a tablet computer to confirm eligibility. Of the 531 eligible patients, 29 women were discharged from medical care before they could be approached, 16 could not be approached in private (e.g., they had a family member present), and 20 declined to participate. Thus, 466 of 486 patients who were approached (96%; 88% of eligible women) consented to participate.

### Measures

#### Race and ethnicity

Race and ethnicity were assessed separately. Ethnicity was assessed with a single question: Do you consider yourself to be Hispanic/Latina? Yes/No. Race was assessed with the following question: “Which of the following best describes your background (select as many that apply): American Indian or Alaska Native; Asian; Native Hawaiian or Other Pacific Islander; African-American or African-American; White or Caucasian; Unknown or I don’t want to answer.”

#### Sexual behavior

One item ascertained relationship status (single, not in a relationship; partnered but unmarried; married; divorced; widowed; other), which was recoded to reflect whether participants were currently single or in a relationship. Participants who identified as divorced, widowed, or other (*n* = 12) were excluded as we could not identify relationship status.

Five items assessed sexual behavior, namely: (c) number of male sex partners (last 3 months), (d) number of male sex partners (lifetime), (e) number of times they had an STI (lifetime), (f) condom use frequency during vaginal and anal sex (last 3 months; “In the past 3 months, when you have had vaginal or anal sex, about how often was a condom used?”; 0 = “Never”; 1 = “Some of the time”; 2 = “Most of the time”; 3 = “Always (100% of the time)”). Number of male sex partners consisted of an open response field.

#### Substance use

Items assessed (a) typical weekly alcohol use (number of drinks) and (b) peak daily alcohol use (last 3 months; “What was the most drinks you drank in a single day?”). Given the non-normal distribution of the peak daily alcohol use variable, and in order to better reflect risk, this variable was used to create two variables reflecting heavy use: binge drinking (i.e., ≥4 drinks) and high-intensity drinking (≥8 drinks on one occasion; [24]). Women’s reported use was dichotomized to reflect whether they engaged in binge drinking and high-intensity drinking.

We also assessed frequency of (c) cigarette, (d) e-cigarettes, (e) water pipe (hookah) for tobacco, and (f) marijuana use on a 6-point Likert-type scale (0 = “never used”, 1 = “have used, but not in the last 30 days”, 2 = “1–9 days”, 3 = “10–19 days”, 4 = “20–29 days”, 5 = “used every day”). Responses to (c)-(f) were dichotomized to reflect current users (used in the last 30 days) versus non-users.

#### Mental health

We assessed depressive symptoms using The Patient Health Questionnaire-2 (PHQ-2: [25]), a well-validated measure designed to assess depressive symptoms in a medical setting. Kroenke et al. [25] determined that a cutoff score of 3 on the PHQ-2 measure is an optimal cut point to detect clinically elevated depressive symptoms within an ambulatory healthcare setting.

#### Health services

Women indicated their interest in receiving information about seven health concerns (i.e., sleep; alcohol use; smoking; marijuana use; relationships and sexual health; coping with stress or anxiety; coping with depression). The survey asked women to indicate: “how interested you are in receiving information on the topic as it relates to your health” on a 4-point Likert-type scale (0 = “Not at all interested” to 3 = “Very interested”).

### Data analytic strategy

Regression analyses were performed to evaluate women’s health behaviors and health needs. Linear regression examined continuous outcomes (i.e., condom use frequency; interest in health topics), Poisson regression examined count outcomes (i.e., number of sexual partners; number of STIs; number of alcoholic drinks; number of cigarettes), and logistic regression examined dichotomous outcomes (i.e., current substance use). In order to understand the intersection of substance use, particularly alcohol and marijuana use, and sexual risk behaviors, we also examined lifetime STIs and number of sex partners accounting for substance use variables. All regression analyses controlled for age. The regression analyses for condom use controlled for relationship status as partner type is associated with condom use [26].

Outliers (i.e., cases with *z* scores ≥3.29) were then recoded as one unit larger than the next most extreme score [27]. There were 10 outliers on lifetime sex partners, corresponding to women reporting more than 50 partners, two outliers on number of partners in the last three months, corresponding to two women who reported more than 15 partners, and 20 outliers on number of weekly drinks, corresponding to women who reported 14+ drinks per week.

## Results

### Participant characteristics

Participants (*N* = 466) ranged in age from 18 to 29 years old (*M* = 23, SD = 3.3). The majority (97%) had at least a high school degree or its equivalent, 28% had a bachelor’s degree or higher, and 43% were part- or full-time students. One-fifth (19%) were unemployed.

One woman did not respond with her race/ethnicity, and 6 (1%) responded “unknown or decline.” One-quarter (25%) identified as Hispanic/Latina. Hispanic individuals in the U.S. often consider their Hispanic identity to be a race when answering separate “race” and “ethnicity” questions [28] as we did in this study. In line with these findings, 61 Hispanic-identified women either reported their racial identity as “unknown” or did not report a racial identity. Regarding only the racial identity question, most of the sample identified as White only (58%). However, when examining both race and ethnicity questions, 53% of the total sample identified as non-Hispanic White. Smaller proportions of women identified as African American (19%), Asian (5%), American Indian (3%), or Native Hawaiian or Pacific Islander (1%). These rates are generally consistent with the most recent census data of the urban area in which the medical clinic is located, although a smaller proportion of the sample identified as non-Hispanic White (53%) compared to the surrounding area (63%: [29]).

Based on answers to these two questions, two sets of dummy coded variables were computed reflecting: non-Hispanic White (*n* = 247) / Hispanic women (*n* = 115); and non-Hispanic White (*n* = 247) / African-American women (*n* = 88). There were too few women in other racial identity categories to create meaningful contrasts, and these women were excluded from further analyses.

### Health behaviors

#### Sexual behavior

All women reported partnered sexual activity (lifetime). Most women were single (31%) or unmarried and in a relationship (60%); 6% were married. Over their lifetime, women had a mean number of 10 male sex partners (median = 6; range = 1–51). A total of 31% had a lifetime history of a STI: One-fifth (21%) of women had one STI, 8% reported having two STIs, and 3% had three or more STIs. The modal number of male sexual partners in the last three months was one (73%), with smaller proportions of women reporting zero partners (3%), two partners (11%), three partners (5%), and four or more partners (7%). In the last 3 months, women who reported having at least one male sexual partner reported that they used condoms “some of the time” (*M =* 1.00, *SD =* 1.01), with single, not partnered women reporting more condom use (*M =* 1.34, *SD =* 1.01) than women in a relationship (*M =* 0.84, *SD =* 1.04).

Compared to non-Hispanic White women, African-American (IRR = 0.91, 95%CI [0.70, 1.19]) and Hispanic women (IRR = 0.89, 95%CI [0.70, 1.13]) did not differ in condom use frequency in the last 3 months controlling for relationship status. In contrast, compared to non-Hispanic White women, Hispanic and African-American women reported significantly fewer lifetime male sexual partners (Hispanic: IRR = 0.53, 95%CI [0.49, 0.58]; African-American: IRR = 0.66, 95%CI [0.60, 0.72]). Because the majority of women had one male partner in the last 3 months and only 13 women reported no sexual partners, racial/ethnicity comparisons were examined using a binary variable reflecting 0–1 versus two or more male sexual partners. Controlling for relationship status, Hispanic women were less likely to report two or more male sex partners in the last 3 months (OR = 0.51, 95%CI [0.27, 0.96]); there was no difference between White and African-American women (OR = 0.69, 95%CI [0.37, 1.32]).

#### STI rates

Hispanic women (IRR = 1.78, 95%CI [1.27, 2.49]) and African-American women (IRR = 2.62, 95%CI [1.87, 3.68]) had a significantly greater risk of STIs than White women. Compared to 27% of White women who had an STI in their lifetime, 40% of African-American women and 36% of Hispanic women had had an STI.

#### Substance use

The majority of women (72%) used alcohol weekly in the last 3 months, with more than one-third (37%) reporting binge drinking and 9% reporting high-intensity drinking. Compared to White women, African-American (IRR = 0.63, 95%CI [0.54, 0.73]) and Hispanic women drank fewer drinks per week (IRR = 0.62, 95%CI [0.54, 0.71]) and were less likely to binge drink (African-American: OR = 0.44, 95%CI [0.25, 0.76]; Hispanic: OR = 0.47, 95%CI [0.29, 0.77]). They did not differ in odds of high-intensity drinking (African-American: OR = 0.47, 95%CI [0.17, 1.28]; Hispanic: OR = 0.71, 95%CI [0.85, 1.05]).

Most women had used marijuana in their lifetime (58%) Table 1. In the last month, 37% used marijuana and 33% reported some form of tobacco use (22% used cigarettes, 4% used e-cigarettes, and 12% used hookah).

**Table 1:** Substance use frequency in the last month

	Never used	Used, but not in last 30 days	1–9 days	10–19 days	20–29 days	Used every day
Marijuana	42%	22%	15%	5%	5%	10%
Cigarettes	68%	11%	7%	2%	3%	10%
E-cigarettes	87%	9%	3%	< 1%	< 1%	1%
Tobacco pipe or hookah	67%	20%	10%	1%	1%	< 1%

African-American (OR = 0.05, 95%CI [0.01, 0.23]) and Hispanic women (OR = 0.19, 95%CI [0.09, 0.39]) were less likely to be current cigarette smokers than White women. They did not differ in current e-cigarette or marijuana use; however, African-American (OR = 5.98, 95%CI [2.53, 14.15]) and Hispanic women (OR = 8.79, 95%CI [4.12, 18.75]) were more likely than White women to smoke tobacco using a water pipe. Among non-Hispanic White women, 34% smoked cigarettes and 4% smoked tobacco using a water pipe or hookah in the last month. In contrast, 8% of Hispanic women and 2% of African-American women smoked cigarettes, but 29% of Hispanic women and 20% of African-American women smoked tobacco using a water pipe in the last month. Regarding any past-month tobacco use (i.e., cigarettes, e-cigarettes, water pipes), non-Hispanic White women (37%) were more likely to be current smokers than African-American women (21%; OR = .50, 95% CI [0.28, 0.91]), but not Hispanic women (34%; OR = 1.05, 95% CI [0.98, 1.12]).

### Mental health

One-in-five (19%) of the young adult women exceeded the threshold on the PHQ-2 indicating clinically significant depressive symptoms. Hispanic women were more likely to report clinically significant depressive symptoms than non-Hispanic White women (OR = 1.95, 95%CI [1.13, 3.38]), but there were no significant differences between African-American and non-Hispanic White women (OR = 0.92, 95%CI [0.46, 1.84]). One-quarter (26%) of Hispanic women had a positive PHQ-2 screen compared to 16% of non-Hispanic White women and 15% of African-American women.

### Self-reported preferences for services

Regardless of race/ethnicity, women expressed the greatest interest in information on coping with stress/anxiety, sleep, and relationships/sexual health, and less interest in substance use (Table 2). When restricted to women who used substances, 57% of women who binge drank, 43% of women who used marijuana, and 67% of women who used tobacco reported they were “not at all interested” in information on alcohol, marijuana, and tobacco use, respectively. Controlling for age, African-American and Hispanic women expressed greater interest in receiving information about sleep (Hispanic: B = 0.31, *p* < .05; African-American: B = 0.39, *p* < .01) and relationships and sexual health (Hispanic: B = 0.24, *p* = .05; African-American: B = 0.46, *p* = .001) compared to non-Hispanic White women. There were no racial or ethnic differences in interest for the other health-related topics.

**Table 2:** Women’s Average Level of Reported Interest in Receiving Information on The Following Health Topics. Topics Ordered by Degree of Interest

		Mean Interest
Coping with stress or anxiety	Non-Hispanic White (*n =* 247)	1.42 (1.12)
	Hispanic (*n =* 115)	1.54 (1.21)
	African-American (*n =* 88)	1.69 (1.24)
Sleep**	Non-Hispanic White	1.11 (1.08)
	Hispanic	1.46 (1.20)
	African-American	1.58 (1.20)
Relationships and sexual health**	Non-Hispanic White	1.02 (1.07)
	Hispanic	1.32 (1.16)
	African-American	1.56 (1.20)
Coping with depression	Non-Hispanic White	0.93 (1.11)
	Hispanic	1.17 (1.26)
	African-American	1.23 (1.23)
Marijuana	Non-Hispanic White	0.48 (0.88)
	Hispanic	0.63 (0.98)
	African-American	0.78 (1.12)
Alcohol	Non-Hispanic White	0.44 (0.80)
	Hispanic	0.52 (0.87)
	African-American	0.61 (0.93)
Smoking, tobacco	Non-Hispanic White	0.27 (0.70)
	Hispanic	0.32 (0.73)
	African-American	0.28 (0.69)

**p < .01. Significance levels refer to significant mean differences (chi-square values) in interest levels between White and non-White women

## Conclusions

Reproductive health clinics often serve as the primary medical home for young women, who are at elevated risk for STIs, unintended pregnancy, and alcohol misuse. Integrated care models can address and provide treatment for behavioral health concerns within a medical setting, improving mental health, patient satisfaction, and other outcomes [30]. Consistent with this, we surveyed young adult women in a reproductive health clinic to assess substance use and sexual behavior and to explore women’s interest in receiving a range of potential behavioral interventions. We found that most women were sexually active with a single partner, used condoms infrequently, drank alcohol in a typical week, and had used marijuana.

Although African-American and Hispanic women reported fewer recent and lifetime sexual partners, no differences in condom use frequency, and less alcohol use than White women, they were more likely to have had an STI, and to have had multiple STIs, in their lifetimes. This increased risk for STIs among minority women is consistent with research [3], and confirms that targeted services continue to be needed to support this population of women specifically. According to leading practitioners [31], STI disparities among ethnic minority women are in part maintained due to poorer access to health care as well as mistrust in the medical system. Therefore, it is critical to better understand the health wishes of diverse young women. Importantly for intervention acceptability, young adult African-American and Hispanic women expressed greater interest in information about sexual health and relationships compared to White women presenting for medical care.

### Mental health needs

Almost one-in-five (19%) women reported depressive symptoms that exceeded the clinical cutoff score. Further, Hispanic women were particularly vulnerable to experiencing depressive symptoms in this sample, with one-quarter reporting clinically significant levels of depressive symptoms. Research suggests that African-American and Hispanic adults are less likely to receive any treatment for depression [21], which is concerning given the equivalent or markedly higher rates of depressive symptoms among women in this sample. These findings highlight the importance of addressing minority mental and behavioral health within medical settings in a culturally sensitive way.

### Substance use

The women in our sample appear to be representative of the overall population of 18–29-year-old young adults in the state in terms of prevalence of use [32]. Nevertheless, a sizable proportion of women evidenced problematic substance use. Over one-third binge drank in the last three months, 9% engaged in high-intensity drinking (i.e., 8+ drinks in one episode), and 10% of women used marijuana daily in the last month. Regarding marijuana use, Hasin et al. [33] found that 21% of 18–29-year old adults in the United States used marijuana in the last year. In contrast in this sample, 35% of this sample of 18–29-year-old women used marijuana in the last month, and 10% used marijuana every day. This greater use might in part reflect regional differences in use, as Hasin et al. found higher rates of past-year use in the northeastern U. S.; however, it might also reflect the needs of the population of young women who seek services at community reproductive clinics. In general, increasing rates of marijuana use in the U. S. are associated with marked increases in marijuana use disorders [33]. Less is known about the impact of frequent marijuana use specifically among women this age; however, some research suggests that marijuana use in the context of romantic relationships increases the risk of unprotected sex [34]. Therefore, this survey highlights this as an important direction for research and, perhaps, intervention.

Further, these findings highlight high rates of tobacco use among young adult women presenting for medical care, and also suggest that focusing on cigarette use might obscure minority women’s risk of tobacco use in these settings. Research has suggested that Hispanic women have the lowest smoking rates of any racial or ethnic group in the United States [35]; however, our findings did not support this finding and instead suggest it is important that providers assess for tobacco use beyond cigarette use in this population, as non-White women in this sample engaged in water pipe tobacco use at rates substantially higher than in the general population of young adults [36]. This is particularly concerning given that individuals perceive waterpipe use to be less addictive and harmful [37], and suggests that providers should include tobacco products in general when screening for tobacco use among patients.

Of note, women in general reported relatively low levels of interest in all health topics, such that only coping with stress or anxiety reached the midpoint of the assessment scale, indicating slight-to-moderate interest. Even though a large proportion of young women reported problematic substance use, there was, overall, little interest in receiving intervention services about their alcohol and other substance use. Although women who engaged in heavy drinking reported greater interest in receiving information on their alcohol use, the majority of women who binge drank still reported they were “not at all interested.” These findings highlight the importance of universal screening for mental health and substance use given that women are unlikely to seek out support in these areas. Universal screening could follow the well-established SBIRT (Screening, Brief Intervention, and Referral to Treatment) model, which empowers healthcare providers to identify substance use and mental health concerns and engage patients in treatment that results in healthcare savings and patient benefit [38].

In addition to universal screening, the results highlight other novel mechanisms for treatment delivery. Based on the association between alcohol use and sexual health, as well as women’s relatively higher interest in their sexual health, there is a need to develop programs that target the co-occurrence of alcohol use and sexual behavior among women. These services would need to be offered in a way that respects the complex (and valued) role that alcohol plays in the social lives of young women and does not scare away women who do not see themselves as needing such services. Such services should also prioritize education around heavy drinking specifically. Fortunately, there are now well-developed interventions approaches (e.g., brief interventions using motivational interviewing) that can be implemented in primary care settings that are well-received by adults who do not see the need for “alcohol treatment” [22].

### Strengths and limitations

Study strengths include our large sample, use of well-established survey items, unique focus on the role of alcohol use in the context of reproductive health care, and high participation rate. Our survey was brief to optimize participation (and local site representativeness), minimize respondent burden, and obtain complete data. In this regard, our approach was successful as 88% of eligible women were screened, and only 20 women (4% of the 531 eligible) declined to be screened. These participation rates are quite strong.

Study limitations include its cross-sectional design, which precludes causal inferences. In addition, as with any single site study, the study location and participant characteristics may not be representative of other locations. For example, almost all participants (97%) had a high school education. The census sample of persons aged 25+ in the same urban region revealed that 24% had less than a high school degree [29]. Thus, study participants were more likely to have obtained a high school degree or its equivalent compared to the local population. To address this limitation, it is important to consider how best to reach women of lower education levels, who often have poorer access to health care [39]. A third limitation is that use of a brief survey prevented a more comprehensive assessment of health behaviors. It is unfortunate that we did not assess sleep and stress management given participants’ interest in interventions related to these topics. Finally, we assessed Hispanic ethnicity broadly without further subgroup assessment. However, the Hispanic population is a heterogeneous, and there are significant differences in health behaviors and needs within this population of women [40]. Moreover, this study focused on sexual activity and condom use with male partners, and so these findings do not shed light on the needs of women who have sex with women, who evidence unique risks [41].

## Conclusions

Young women at sexual and reproductive healthcare centers report sexual risk behavior and substance use, singly and together, in ways that can increase risk for STIs, unintended pregnancy, and other adverse health outcomes. Sexual risk behavior and substance use levels among women at these settings are often elevated compared to similar aged women in the general population. Young women, particularly non-White women, appear receptive to receiving information and services targeted to sexual and relationship health as well as to other health concerns (e.g., sleep, stress). Interest in substance use services is less strong, indicating the need for substance counseling services that are not stigmatizing given the high rates of substance use and misuse in this population of young adult women. The present results indicate that reproductive healthcare centers provide opportune settings in which to address the often unmet behavioral health needs of young women.

## Abbreviations

HPV: Human papillomavirus
PHQ-2: Patient Health Questionnaire-2
STI: Sexually transmitted infections

## References

[1] STDs in adolescents and young adults—2016 STD surveillance report. Centers for Disease Control and Prevention 2016. https://www.cdc.gov/std/stats16/adolescents.htm. Accessed 28 Mar 2018.

[2] Satterwhite CL, Torrone E, Meites E, Dunne EF, Mahajan R, Cheryl Bañez Ocfemia M (2013). Sexually transmitted infections among US women and men: prevalence and incidence estimates, 2008. Sex Transm Dis.

[3] STDs in racial and ethnic minorities—2016 STD surveillance report. Centers for Disease Control and Prevention 2016. https://www.cdc.gov/std/stats16/minorities.htm. Accessed 13 Feb 2018.

[4] Finer LB, Zolna MR (2016). Declines in unintended pregnancy in the United States, 2008–2011. N Engl J Med.

[5] Sinozich S, Langton L. Rape and sexual assault victimization among college-age females, 1995–2013. US Department of Justice. 2014; https://www.bjs.gov/content/pub/pdf/rsavcaf9513.pdf. Accessed 2 Mar 2018.

[6] Rosser ML, Brusati AJ (2014). An opportunity for obstetrician-gynecologists to affect the epidemic of cardiovascular disease in women. Obstet Gynecol.

[7] Stidham Hall K, Moreau C, Trussell J (2011). Discouraging trends in reproductive health service use among adolescent and young adult women in the USA, 2002-2008. Hum Reprod.

[8] Carey MP, Carey KB, Maisto SA, Gordon CM, Schroder KE, Vanable PA (2004). Reducing HIV-risk behavior among adults receiving outpatient psychiatric treatment: results from a randomized controlled trial. J Consult Clin Psychol.

[9] Fisher JD, Fisher WA, Cornman DH, Amico RK, Bryan A, Friedland GH (2006). Clinician-delivered intervention during routine clinical care reduces unprotected sexual behavior among HIV-infected patients. J Acquir Immune Defic Syndr.

[10] Racial/Ethnic differences in mental health service use among adults. 2015. https://www.integration.samhsa.gov/MHServicesUseAmongAdults.pdf. Accessed 20 Mar 2018.

[11] Scott-Sheldon LAJ, Carey KB, Cunningham K, Johnson BT, Carey MP (2016). Alcohol use predicts sexual decision-making: a systematic review and meta-analysis of the experimental literature. AIDS Behav.

[12] Kanny D, Liu Y, Brewer RD, Lu H (2013). binge drinking - United States, 2011. Morb Mortal Wkly Rep.

[13] Kann L, McManus T, Harris WA, Shanklin SL, Flint KH, Hawkins J, et al. Youth risk behavior surveillance: United States, 2015 Morb Mortal Wkly Rep 2016;65. https://www.cdc.gov/healthyyouth/data/yrbs/pdf/2015/ss6506_updated.pdf. Accessed 28 Mar 2018. 10.15585/mmwr.ss6506a1 27280474

[14] Graves KL (1995). Risky sexual behavior and alcohol use among young adults: results from a national survey. Am J Health Promot.

[15] Caetano R, Baruah J, Ramisetty-Mikler S, Ebama MS (2010). Sociodemographic predictors of pattern and volume of alcohol consumption across Hispanics, blacks, and whites: 10-year trend (1992-2002). Alcohol Clin Exp Res.

[16] Christie-Mizell C, Laster Pirtle W, Tyndall BD, Merolla DM (2015). Race-gender differences in the impact of history of heavy drinking on current alcohol consumption during the transition to adulthood. J Sociol Soc Welf.

[17] Mojtabai R, Olfson M, Han B (2016). National trends in the prevalence and treatment of depression in adolescents and young adults. Pediatr..

[18] Khan MR, Kaufman JS, Pence BW, Gaynes BN, Adimora AA, Weir SS (2009). Depression, sexually transmitted infection, and sexual risk behavior among young adults in the United States. Arch Pediatr Adolesc Med.

[19] Lehrer JA, Shrier LA, Gortmaker S, Buka S (2006). Depressive symptoms as a longitudinal predictor of sexual risk behaviors among US middle and high school students. Pediatr.

[20] Brody DJ, Pratt LA, Hughes JP (2018). Prevalence of depression among adults aged 20 and over: United States, 2013-2016. NCHS Data Brief.

[21] Alegría M, Chatterji P, Wells K, Cao Z, Chen C, Takeuchi D (2008). Disparity in depression treatment among racial and ethnic minority populations in the United States. Psychiatr Serv.

[22] Moyer VA (2013). Screening and behavioral counseling interventions in primary care to reduce alcohol misuse: U.S. preventive services task force recommendation statement. Ann Intern Med.

[23] Pascale A, Beal MW, Fitzgerald T (2016). Rethinking the well woman visit: a scoping review to identify eight priority areas for well woman Care in the era of the affordable care act. Women’s Heal Issues.

[24] Patrick ME, Azar B. High-intensity drinking. Alcohol Res. 2017;39(1). 10.35946/arcr.v39.1.08 PMC6104968 30557148

[25] Kroenke K, Spitzer RL, Williams JBW (2003). The patient health Questionnaire-2. Med Care.

[26] Macaluso M, Demand MJ, Artz LM, Hook EW (2000). Partner type and condom use. AIDS.

[27] Tabachnick BG, Fidell LS (2013). Using multivariate statistics.

[28] Hitlin S, Elder JSB, GH J (2007). Measuring Latinos: racial vs. ethnic classification and self-understandings. Soc Forces.

[29] QuickFacts: Providence city, Rhode Island. United States Census Bureau. https://www.census.gov/quickfacts/fact/table/providencecityrhodeisland/EDU635216#viewtop. Accessed 4 Apr 2018.

[30] Reed SJ, Shore KK, Tice JA (2016). Effectiveness and value of integrating behavioral health into primary care. JAMA Intern Med.

[31] Douglas J, Kevin Fenton C, Aral SO, Adimora A, Tyler-Hill Y, Keppel KG, et al. Consultation to address STD disparities in African-American communities. Centers for Disease Control and Prevention. 2007; https://www.cdc.gov/std/general/stdhealthdisparitiesconsultationjune2007.pdf. Accessed 20 Mar 2018.

[32] Rosenthal S. Substance use and mental health in Rhode Island (2015): A state epidemiological profile. 2015. http://www.riprc.org/wp-content/uploads/2016/04/2015_RI_State_Epi_Profile_Final_2-2016_rev.1.pdf. Updated February 29, 2015. Accessed 29 Mar 2018.

[33] Hasin DS, Saha TD, Kerridge BT, Goldstein RB, Chou SP, Zhang H (2015). Prevalence of marijuana use disorders in the United States between 2001-2002 and 2012-2013. JAMA Psychiatry.

[34] Walsh JL, Fielder RL, Carey KB, Carey MP (2014). Do alcohol and marijuana use decrease the probability of condom use for college women?. J Sex Res.

[35] Tobacco product use among adults—United States, 2012–2013 (2014). Centers for Disease Control and Prevention. Morb Mortal Wkly Rep.

[36] Majeed BA, Sterling KL, Weaver SR, Pechacek TF, Eriksen MP (2017). Prevalence and harm perceptions of hookah smoking among U.S. adults, 2014–2015. Addict Behav.

[37] Primack BA, Sidani J, Agarwal AA, Shadel WG, Donny EC, Eissenberg TE (2008). Prevalence of and associations with waterpipe tobacco. Ann Behav Med.

[38] Hargraves D, White C, Frederick R, Cinibulk M, Peters M, Young A (2017). Implementing SBIRT (screening, brief intervention and referral to treatment) in primary care: lessons learned from a multi-practice evaluation portfolio. Public Health Rev.

[39] Executive summary (2009). Women and health. World Health Organization.

[40] Ai AL, Appel HB, Huang B, Lee K. Overall health and healthcare utilization among Latino American women in the United States. 2012;21(8):878–885. doi:10.1089/jwh.2011.3431. 10.1089/jwh.2011.3431 22747245

[41] Everett BG (2013). Sexual orientation disparities in sexually transmitted infections: examining the intersection between sexual identity and sexual behavior. Arch Sex Behav.

